# the contribution of south Africans to the subject of dilated cardiomyopathy

**Published:** 2009-02

**Authors:** David A Watkins, Bongani M Mayosi

**Affiliations:** Duke University School of Medicine, Durham, North Carolina, USA, and Research Fellow, Department of Medicine, University of Cape town, Observatory; Department of Medicine, Groote Schuur Hospital and University of Cape town, Observatory

## Abstract

**Background:**

Dilated cardiomyopathy (DCM) is a heart muscle disease that is endemic in Africa. Over the past 50 years, South African investigators have made significant contributions to scientific elucidation of the condition. The objective of this review was to summarise their research on the subject of DCM.

**Methods and results:**

We searched PubMed for articles originating from South Africa and focusing on DCM or the related condition, peripartum cardiomyopathy (PCM). Reference lists and prominent South African researchers on DCM were also consulted. The prevalence of DCM is comparable in magnitude to that of other endemic heart conditions such as hypertension and rheumatic heart disease, although by comparison, DCM may cause disproportionate morbidity from heart failure. In the African context, malnutrition, excessive alcohol intake, prior myocarditis and genetic make-up have been proposed as aetiologies, and some or all of these factors may play an interrelated role in individual disease expression. The pathogenesis of DCM is partially due to the mechanical effects of fibrosis, and the immune response to myocardial damage likely affects disease progression. Small trials of pentoxifylline plus conventional therapy have demonstrated a trend towards reduced mortality from heart failure.

**Conclusions:**

Despite half a century of noteworthy research, the pathogenic mechanisms of DCM are still incompletely understood. South Africans have, however, played and should continue to play a critical role in advancing research on DCM.

## Summary

As in industrialised countries, heart failure is an important cause of morbidity and mortality in developing countries. In the latter settings, however, heart failure arises primarily from non-ischaemic causes, such as hypertension, rheumatic heart disease and cardiomyopathy.^1^ Idiopathic dilated cardiomyopathy (DCM), a disease of heart muscle that leads to cardiac dysfunction and heart failure, is endemic in Africa. DCM has been recognised in various forms for more than half a century, but much is still unknown about its cause and true prevalence on the continent.^2^ However, despite relatively scant attention from the international community, much has been done to elucidate the nature of the disease. Since DCM was first described in South Africa,^3^ investigators in this country have played a critical role in advancing scientific understanding of the condition.

The primary aim of this article is to summarise the contributions of South African research in the field of DCM. We include research dealing with peripartum cardiomyopathy (PCM), because PCM is regarded by some authorities to be a variant of idiopathic DCM, although others consider it to be a separate entity.^4^ From an historical standpoint, what is now called DCM was for many years termed ‘congestive cardiomyopathy’,^5^ and before that, was given a myriad other names.^6^ For the purpose of clarity, we refer to older literature using the currently accepted DCM where it is clear that this reflects the disease being discussed. Studies dealing with pregnancy-associated heart failure of unknown aetiology are referred to as PCM.

We searched PubMed using the terms *cardiomyopathy, dilated cardiomyopathy*, and *congestive cardiomyopathy*, with and without the modifiers *Africa* and *South Africa*. Reference lists from relevant articles were reviewed. We also consulted several of the original South African investigators of DCM and PCM. Only studies published in English and with a first or last author based in South Africa have been included.

## Historical perspective

The earliest reports of DCM in South Africa were small clinical or pathological case series, mostly of black patients in the Johannesburg area. In 1951, Gillanders reported 30 cases of unexplained congestive heart failure among urban black patients presenting to the Chris Hani-Baragwanath Hospital,^3^ and the next year he and colleagues reported another 12 patients with similar symptoms.^7^ Becker and colleagues, in a 1953 autopsy series,^8^ described 40 cases of rapidly progressive heart failure that they believed represented a unique pathological entity. By the early 1960s, several similar case series had been published.^9^-^12^ The emphases of these reports were on different clinical and pathological features, however, and there was considerable controversy among researchers as to whether or not these reports were describing the same disease.^13^

By contrast, PCM had been described in other parts of the world decades before it was reported in Africa.^4^ Its first descriptions in Africa were by Seftel and Susser in Johannesburg,^14^ then by Reid in Durban,^15^ both in 1961. Of these individuals, Seftel would later write extensively on the aetiology and treatment of DCM, as well as PCM. In total, however, there were relatively few early reports of PCM compared to DCM, and although its aetiology was also unknown, the disease did not generate the same degree of controversy in the literature.^13^

Complicating early research and discussions about DCM were conflicting systems of nomenclature and classification. South African physicians had a unique role in both the origin of these systems and the evolution of a more standardised terminology. In a 1968 review of the literature from South Africa, Böhm found 27 different terms for what he called ‘South African endomyocardiopathy’. The variety of terms proposed – some even by the same authors in different publications – reflected a lack of knowledge about its aetiology and relation to other causes of heart failure.^6^

The specific term ‘cardiomyopathy’ came into favour over several years, between the time of Brigden (1957) and the International Symposium on Cardiomyopathies,16 which was held in 1971 in Tiervlei, South Africa.^17^ This symposium reviewed the most current international research on cardiomyopathy, including work from prominent South Africans. It was also organised in part by the South African Medical Research Council and cochaired by Andries Brink from the University of Stellenbosch.^17^ Unfortunately, a standardised nomenclature was not agreed upon until a decade later.^18^

## Epidemiology

The true burden of DCM and PCM in Africa is unknown. In contrast to more industrialised regions, there have been no population-based studies to assess its true prevalence on this continent.^2^ It is widely accepted that cardiomyopathy is endemic, particularly among black Africans,^19^ but unfortunately, the sizes of the studies that have been performed and their ethnic composition, and therefore their estimates of disease burden, vary considerably.

Unfortunately, only one study from South Africa has estimated the frequency of PCM specifically. Based on 97 patients seen over four years, its incidence was proposed to be one in 1 000 deliveries.^20^ On the other hand, a number of case series from South Africa suggest a heavy burden of DCM; Table 1 lists the relevant data from these studies. In clinical series, DCM accounted for 11.6 to 37.5% of diagnoses of heart disease^10^,^11^,^21^ and 15.4 to 48% of admissions for heart failure.^22^-^24^ In necropsy series, DCM was the cause of death in 14.1 to 17% of cases of heart disease^25^,^26^ and 12.7% of deaths from heart failure.^27^ In comparison, a review of 39 408 electrocardiograms in Cape Town, mostly from white and coloured patients, found DCM in fewer than 1% of these tracings,^28^ demonstrating ethnic variation in the prevalence of this disease.

**Table 1:** Proportions Of DCM Cases In Studies Included In This Review

*Study ID*	*Year*	*Patient population*	*Method of diagnosis*	n	*% of CVD diagnoses*	*% of HF admissions*	*% of CVD deaths*	*% of HF deaths*
Schwartz *et al.*^10^	1958	Series of CVD admissions	clinical	275	37.5	-	-	-
Cosnett^11^	1962	Series of CVD admissions	clinical	1000	13.8	-	-	-
McGlashan^21^	1988	Series of CVD admissions	clinical	4618	11.6	-	-	-
Powell and Wright^22^	1965	Series of HF admissions	clinical	270	-	34	-	-
Maharaj^23^	1991	Series of HF admissions	clinical	225	-	48.4	-	-
Sliwa *et al.*^24^	2008	Series of HF admissions	echocardiogram	1593	-	15.4	-	-
Isaacson^25^	1977	Series of CVD deaths	autopsy	120	-	-	14.1	-
Steenekamp^26^	1992	Series of CVD deaths	autopsy	90	-	-	17	-
Kallichurum^27^	1969	Series of HF deaths	autopsy	857	-	-	-	12.7

CVD: cardiovascular disease; HF: heart failure; %: percentage.

Therefore the frequency of diagnosis of DCM, especially at hospital admission for heart failure, is relatively high, while the rate of death from DCM, especially compared with other cardiovascular diseases, e.g. hypertensive or rheumatic heart disease, is still relatively low. These data imply that compared to other conditions, DCM carries a disproportionate share of the morbidity from heart disease.

## Aetiology

Perhaps the most perplexing aspect of DCM is its aetiology. Much has been written on this subject over the past 50 years, yet its precise cause is still unknown. Currently, it is held that DCM represents a ‘final common expression’ of a wide variety of insults, rather than the effects of one causative agent.^2^ The most important contributions from South Africa include studies on the roles of nutrition, alcohol, prior myocarditis, and genetics in DCM. Early South African studies on PCM also sought to define its aetiology and risk factors as a unique form of cardiomyopathy.

Nutritional deficiency was the first causative agent proposed for DCM, and most of the early studies focused on the relationship between diet and heart failure. Gillanders’ original work, titled ‘Nutritional heart disease’, postulated a connection between an urban ‘Bantu’ diet deficient in certain vitamins and protein, and the development of heart failure.^3^ A follow-up study argued that haemosiderosis and hepatic fatty changes, seen universally in pathological specimens, implied a nutritional cause.^7^ Seftel also recognised the possible role of malnutrition in DCM, though his hypotheses also invoked other causes.^29^ Eventually, malnutrition as a sole explanation for DCM fell out of favour, but recently, nutritional iron overload was revisited as a potential cause of DCM in select patients.^30^

It must also be noted that beriberi heart disease, which is due to thiamine deficiency, was distinguished early on from ‘idiopathic’ DCM. In the latter, red blood cell thiamine concentrations were shown to be normal, in contrast with those in beriberi.^31^ Beriberi was frequent among hostel-dwelling, migrant labourers who drank heavily and developed acute heart failure.^32^ In contrast to DCM, these patients usually presented with ‘high-output’ heart failure and responded to parenteral thiamine.^33^ On the other hand, some patients with beriberi had persistent cardiomegaly, even after treatment with thiamine, and later reports confirmed that beriberi was sometimes associated with underlying cardiomyopathy. 34

Excessive alcohol intake has been identified in many populations at risk for DCM, but in contrast to nutritional hypotheses, alcohol is still widely accepted as a common cause of DCM.^2^ Grusin’s study was the first in South Africa to make an explicit link between alcohol and cardiomyopathy.^9^ At the International Symposium on Cardiomyopathies, Seftel hypothesised a different role for alcohol in DCM versus beriberi heart disease.^35^ The latter was seen in acute thiamine deficiency, secondary to heavy alcohol use, often in young men; but the former was seen in older individuals with a pattern of chronic use. In this view, alcohol was thought to play a more direct role in the damage of the myocardium of DCM, although over many years.^35^ Seftel’s later writings reiterated these ideas, but he also stressed that DCM was not due to one causative agent, but rather to the interplay of risk factors such as general malnutrition, thiamine deficiency, acute or chronic alcohol use, and patient lifestyle.^29^,^36^,^37^ A later study confirmed alcohol excess, thiamine deficiency and vitamin B_6_ deficiency in many patients with DCM, although beriberi heart disease as a specific entity was relatively rare.^38^

In the 1980s, it was hypothesised that DCM might be a sequela of prior myocarditis, and two studies by Rose and colleagues sought to define the relationship between myocarditis and DCM.^39^ Review of 76 endomyocardial biopsies^5^,^39^ and 54 autopsies^5^ showed frequent degenerative changes and myocyte hypertrophy, but infrequent myocarditis, suggesting that autoimmunity or infectious agents were not likely to be part of the aetiology of DCM.

In the last two decades, one of South Africa’s major contributions to the field of DCM has been in elucidating its genetic basis. Initially, reports were on familial cases, but later more extensive studies were undertaken. Brink and colleagues were the first in South Africa to describe a case of familial DCM with prominent ventricular arrhythmias.^40^ Cases of DCM were also linked to progressive familial heart block types I^41^ and II.^42^ Other reports included DCM in two brothers,^43^ and a case of microcephaly associated with DCM.^44^

More recently, mutation screening of 57 patients demonstrated that actin mutations do not play a major role in dilated cardiomyopathy.^45^ The same investigators later reported that a mitochondrial DNA susceptibility gene increases the risk of sporadic DCM in the general population.^46^ Three studies evaluated the role of mutations in signalling pathway proteins, specifically polymorphisms in the angiotensin-converting enzyme^47^ and beta-adrenoreceptor subtypes,^48^,^49^ in the progression of DCM. Finally, two extensive reviews on the genetics of cardiomyopathy were also recently published.^50^,^51^

Regarding the aetiology of PCM, Seftel and Susser’s original article outlined two early hypotheses, one related to environmental factors and the other to physiological stresses in susceptible patients.^14^ Seftel consistently argued for the latter, suggesting that PCM arises from ‘acute on chronic’ cardiac malnutrition during a physiologically vulnerable state. He later advanced the hypothesis that PCM is merely a form of idiopathic cardiomyopathy. 35 Although little was written about the aetiology of PCM in subsequent decades, two recent reviews have summarised international progress in this field.^4^,^52^

## Pathogenesis

Important contributions dealing with the pathogenesis of DCM and PCM have also come from South Africa. During the 1960s and 1970s, research focused primarily on the biochemical and metabolic effects of DCM on the myocardium and its mechanical and structural characteristics. Since the 1990s, however, investigators have written mostly on the relationship between myocardial damage and immunity.

Brink and colleagues quantified myocardial blood flow and metabolism^53^ as well as mechanical function and compliance^54^ in DCM, and they compared these findings to other forms of heart disease. Taken with later cine-angiographic data,^55^ they concluded that heart failure in DCM is related more to the mechanical effects of fibrosis than to metabolic or haemodynamic disturbances within the myocardium.

Other investigators found that hypokinesis was universal in DCM, but in contrast to ischaemic heart disease, ‘asynergy’, i.e. non-coordinated myocardial contraction, was variable.^56^ Another study compared simple bedside haemodynamic estimates to angiography and found several sensitive indicators of left ventricular dysfunction.^57^ Much later, improved ventricular function following initiation of heart failure therapy was linked to a polymorphism in the aldosterone synthetase gene, which might explain heritable variations in response to drug therapy.^58^

Immune response to myocardial damage most likely plays a significant role in the progression of DCM, and a variety of inflammatory molecules appear to be involved. One study found that HLA-DR1 and HLA-DRw10 were more common in patients with DCM, suggesting a genetically determined immune response contributes to the pathogenesis of this disease.^59^ Other studies found tumour necrosis factor alpha (TNF-α) and C-reactive protein (CRP) elevations in idiopathic DCM.^60^,^61^ Leukocyte cytokines were also shown to be elevated, even after patients were haemodynamically stabilised.^62^,^63^ These are in contrast to other cytokines, which decrease with treatment.^60^,^61^

A similar inflammatory response was found in PCM, except that Fas/Apo-1 was also elevated;^64^ this marker also predicted mortality in PCM patients.^65^ Additionally, class G3 immunoglobulins were uniquely elevated in PCM versus DCM, implying different effects of PCM on humoral immunity.^66^ Although precise mechanisms are still elusive, these data have begun to influence the pharmacotherapy of DCM and PCM.^67^

## Clinical and pathological features

In spite of its unknown aetiology, DCM has been reported to have distinctive clinical features that contrast with other forms of heart disease. Gillanders noted congestive heart failure with an extreme degree of oedema.^30^ Many patients tended to form emboli and subsequent infarctions, particularly in the viscera.^11^,^68^ Unlike constrictive pericarditis and tamponade, DCM caused no changes in the second heart sound.^69^

In early paediatric reports, DCM was also shown to have similar clinical and pathological characteristics as in adults.^12^ One study found that all patients with DCM had left heart enlargement and hypertrophy. Many of these, especially coloured patients, also had conduction defects.^70^ In another study, 90% of black patients with DCM who were in congestive heart failure were found by telemetry to have ventricular arrhythmias. Over three years, non-sustained ventricular tachycardia was associated with death in 65% of these patients.^71^

Lowenthal^72^ and Mokhobo^73^ reported on patients who had been misdiagnosed with DCM when in fact they had hypertensive heart disease, and these authors discussed the distinction between the two, and warned against over-diagnosis of cardiomyopathy. From a pathological standpoint, endomyocardial biopsy was shown to be useful in the diagnosis of DCM, although morphology often correlated poorly with clinical features.^39^

DCM in Africa was found to carry a lower incidence of associated anaemia and renal failure versus western heart-failure cohorts, most likely due to differences in the prevalence of ischaemic heart disease and treatments that affected renal function.^74^ In support of early descriptions,^14^ PCM was found to be more common in older, multiparous women and those who breastfed for an extended period of time.^20^ Adverse outcomes were associated with late presentation20 and subsequent pregnancy.^75^

## Treatment

While South African researchers have written extensively on conventional treatments for DCM and associated heart failure, they have also contributed to the development – and in many cases success – of alternative therapies as well. Diuretics^33^ and then beta-blockade^76^ were promoted early on and are still the mainstays of therapy. Later studies evaluated a new beta-blocker^77^ and showed that pre-treatment with beta-blockers improved the response to angiotensin-converting enzyme inhibitor therapy.^78^ For heart failure refractory to oral medications, parenteral amrinone and dobutamine were effective as short-term therapy.^79^

Cardiac transplantation is a standard therapeutic option for advanced heart failure and was pioneered in South Africa.^76^ Other therapies that have been proposed include palliative pericardiotomy for refractory heart failure,^80^ and treatment with thiamine and nicotinamide, as well as protein supplementation, to correct deficiencies in these nutrients.^81^ Seftel’s early work also mentioned a possible role for milk powder supplements, as well as prevention of cardiomyopathy by abstaining from alcohol.^33^

Perhaps the most unique contribution of South African investigators in the treatment of DCM was the investigation of pentoxifylline for heart failure. This drug suppresses the immune response to DCM, particularly TNF-α^60^ and CRP.^61^ In the first clinical study, pentoxifylline improved cardiac function over six months of therapy,^60^ and these results were replicated in later studies.^82^-^84^ While pentoxifylline has shown promise, a systematic review of these studies – which have been the only randomised studies on the drug – concluded that although there was a trend towards reduced mortality, the results were not statistically significant, and larger trials were needed.^67^

In general, treatment of PCM has been dealt with similarly to DCM. However the prognosis of PCM is better than DCM, which originally led Seftel to advocate for more aggressive treatment, aiming for full recovery. The variety of drugs available was more limited at the time, so his recommendations focused on optimisation of nutrition, limitation of breastfeeding, and avoidance of future pregnancy.^33^ Later, continuous veno-venous haemofiltration was shown to be effective in PCM patients with severe fluid overload.^85^ Finally, in addition to its benefits in DCM, pentoxifylline along with conventional therapy was shown to be beneficial in PCM.^83^

## Conclusions

Although DCM and PCM are important causes of heart failure in resource-poor settings, much has been accomplished in elucidating the nature of these diseases since they were first described in Africa. The work of South African physicians and scientists in assessing the prevalence, causes, clinical features and treatment of cardiomyopathy is a biomedical success story and should be an inspiration for future research.

This review has shown, however, that work on cardiomyopathy is far from complete. The contributions of genetics and immunology to DCM and PCM are far from clear. Basic research on cardiomyopathy needs to continue, ideally with more international financial support and collaboration. Treatments for cardiomyopathy-associated heart failure are far from optimal, and more rigorous clinical trials will be required to advance the newest therapies.

On a population level, the social determinants of cardiomyopathy are not well understood, and neither is its true epidemiology. Population-based studies and detailed international registries will provide better information on the global burden of disease, and they will facilitate the development of an appropriate multi-national research agenda, as well as the implementation of public health measures to modify environmental risk factors where possible.
